# An evaluation of a mental health literacy course for Arabic speaking religious and community leaders in Australia: effects on posttraumatic stress disorder related knowledge, attitudes and help-seeking

**DOI:** 10.1186/s13033-020-00401-7

**Published:** 2020-08-20

**Authors:** Shameran Slewa-Younan, Maria Gabriela Uribe Guajardo, Yaser Mohammad, Henry Lim, Gabriela Martinez, Randa Saleh, Michele Sapucci

**Affiliations:** 1 Mental Health, Translational Health Research Institute, Macarthur Clinical School, School of Medicine, Western Sydney University, Locked Bag 1797 Penrith, Sydney, NSW 2751 Australia; 2 Honorary Senior Research Fellow, Centre for Mental Health, Melbourne School of Population and Global Health, University of Melbourne, Melbourne, Australia; 3 School of Medicine, Research Officer, Mental Health, Western Sydney University, Sydney, Australia; 4 Mental Wellbeing, Health Promotion Service, Population Health, South Western Sydney Local Health District, Sydney, Australia; 5 Adult Mental Health Team, Bankstown Community Mental Health, South Western Sydney Local Health District, Sydney, Australia; 6 Mental Health Promotion, Prevention & Early Intervention, Transcultural Mental Health Centre, Western Sydney Local Health District, Sydney, Australia

**Keywords:** Refugee, Mental health, Mental health literacy, Mental health promotion, Community and religious leaders, Arabic speaking

## Abstract

**Background:**

Australia is an ethnically diverse nation with one of the largest refugee resettlement programs worldwide, including high numbers of refugees with an Arabic speaking background. Evidence suggests that refugees can demonstrate high levels of psychological distress and are at a higher risk of developing mental illness such as posttraumatic stress disorder (PTSD) and major depressive disorder (MDD). Notwithstanding, research has also shown Arabic speaking refugees have lower levels of professional help-seeking behaviours, postulated to be related to mental health literacy levels.

**Methods:**

A culturally sensitive mental health literacy (MHL) training program was developed and delivered in Arabic to Arabic speaking religious and community leaders using a 1-day training workshop format. An uncontrolled pre-, and post study design was used to provide a preliminary evaluation of improvement in PTSD-related knowledge, attitudes and help-seeking measures.

**Results:**

A total of 54 adults were trained, with 52 completing the pre- and post-intervention questionnaire. Significant differences were found post-training in measures such as the ability to recognise mental health problems (p = 0.035) and an increased recognition of the role that medication can play in the treatment of PTSD (p = 0.00). Further, an improvement in negative attitudes such as a desire for social distance (p = 0.042) was noted and participants reported more helpful strategies in line with promoting professional help-seeking following training (p = 0.032).

**Conclusion:**

Our findings indicated the training led to an improvement of some measures of MHL. To the best of our knowledge, this is the first time that the MHL program has been tailored for Arabic speaking religious and community leaders; who assist refugees with an Arabic background. By equipping community leaders with the knowledge to better respond to mental health problems, the overall goal of improving the mental health outcomes of Arabic speaking refugee communities is closer to being realised.

## Background

The world is facing an unprecedented challenge, with alarmingly high numbers of forcibly displaced persons. Recent figures reported by the United Nations High Commissioner for Refugees (UNHCR) indicate this number to be in the vicinity of 70 million [1]. Disturbingly, the ongoing conflicts in the Middle East have resulted in 10 million displaced people most originating from Syria and Afghanistan alone [1]. Australia has one of the largest resettlement programs worldwide [2] providing a durable solution and protection to individuals through the Humanitarian Visa Program. In the 2018–19 period, the Australian Government allocated 18,750 places to refugees and others who were displaced as a result of conflict, persecution and human rights abuses [3].

Data from the Australian Bureau of Statistics (ABS) indicates that a majority of these ethnically diverse groups choose to resettle in major cities in Australia, and predominately in New South Wales (NSW) (33%) [4]. Relatively, Metropolitan Sydney had the largest overseas-born population of all the capital cities [4], concentrated primarily in South Western Sydney. More specifically, since 2016 approximately 3000 Arabic speaking refugees have chosen to settle in the Local Government Area (LGA) of Fairfield alone. As such South Western Sydney is currently facing an unprecedented challenge in meeting the health and mental health needs of these new arrivals.

Evidence suggests that refugees demonstrate high levels of psychological distress and are at a higher risk of developing mental illnesses such as posttraumatic stress disorder (PTSD) and major depressive disorder (MDD) [5]. Research has also demonstrated that Arabic speaking refugees have lower levels of professional help-seeking behaviours [6–8]. An important concept that may be related to professional help-seeking behaviours is mental health literacy. The term ‘mental health literacy’ (MHL) was introduced by Jorm and colleagues [9] as an extension of the concept of ‘health literacy’. It is defined as “knowledge and beliefs about mental disorders which aid their recognition, management or prevention” [9] and includes: the ability to recognise specific disorders; knowing how to seek mental health information; knowledge of risk factors and causes, of self-treatments and of professional help available; and attitudes that promote recognition and appropriate help-seeking. Australia is a world leader in MHL research and this research has been used, with encouraging results, to inform the conduct of community-based health promotion programs designed to improve public awareness and understanding of mental health issues and facilitate early, appropriate help-seeking among individuals with mental health problems [10]. By contrast, the MHL of culturally diverse communities is an emerging area of research [11]. Specific to this study is the evidence related to Arabic speaking refugee groups, which has demonstrated that differing levels of knowledge and beliefs about the nature and the management of mental health problems may act as barriers to help-seeking [11, 12]. It is postulated that culturally tailored mental health education and promotion programs addressing these barriers are required. Relatedly, research has demonstrated the well-regarded mental health first aid (MHFA) program delivered to culturally diverse groups such as the Chinese and Vietnamese communities in Australia, can lead to improvements in the MHL in these groups following such interventions [13, 14]. Spurred on by such successes, a number of MHFA interventions have since been undertaken within refugee populations including the capacity of building community workers to assist refugees with mental health problems [15] and improving the MHL and help-seeking behaviours of teens from culturally diverse backgrounds and the responsible adults who work with them [16].

## Religious and other community leaders: the need for mental health literacy training to develop mental health allies

Another equally important support group working with refugees are religious and community leaders [17–19]. Importantly, research has demonstrated that clergy in Arabic speaking communities are highly revered and considered to be the first point of contact for people who are suffering from mental illnesses [18]. However, there is also evidence to suggest that such leaders may have poorer knowledge related to the recognition and management of mental illness despite their significant influence [18]. Thus improving their capacity to respond to refugees with mental health problems may play a role in promoting professional help-seeking where it is required.

In a community based study of ethnic minorities residing in south east London, it was noted that Asians and Black Africans were more likely to seek help from religious leaders compared to general population, with rates of 15% and 18% reported respectively [20]. This trend has also been shown to apply to the Arabic community. A qualitative study conducted in Arabic speaking communities in Australia, highlighted religious leaders ‘would be the person of choice’ for advice and counselling in times of distress. Moreover, it was reported that the majority of participants (74%) perceived religious leaders to have spiritual healing powers [18]. Participants noted that approaching religious leaders to help explain and alleviate the confusing circumstances and symptoms arising from a mental illness was viewed to be less threatening than a psychiatrist [18]. However, and despite refugees’ perceived benefits around the role of religious leaders, evidence suggests that such leaders can be poorly equipped to provide effective support to those with mental illness [19]. Moreover, evidence on mental health referral behaviour amongst religious leaders suggests that their knowledge of mental health systems can be problematic, highlighting an area for specific mental health educational interventions [17]. Nonetheless, such interventions are rare, with few intervention programs targeting the MHL of religious and community leaders to assist ethnic minorities with mental health problems [21, 22]. Specifically, Subedi et al. [21] reported on the impact of a 1-day MHFA training program delivered to Bhutanese refugee community leaders based in the United States. A total of 58 participants completed a pre and post- training survey which was a culturally adapted version of the MHL instrument developed for MHFA training in Australia [21]. Surveys were completed immediately prior to and after the MHFA intervention. The assessment included a vignette describing a person suffering depression followed by questions assessing knowledge and attitudes about mental health conditions and questions regarding post-resettlement stressors. Significant improvement was shown in the correct identification of mental health conditions, knowledge of treatment options for the mental health problem in the vignette and confidence relating to the provision of support for individuals suffering from, mental health problems. However, no change was observed for stigmatising attitudes [21]. A second study undertaken in Ghana, sought to measure the impact of a 3-h MHL programme on community leaders’ knowledge about and attitudes toward people with mental disorders using a cluster randomised controlled design [22]. An adapted MHL survey was administered at pre-training and 12-week post-training points. Overall, the findings of the study indicated that using a problem-solving Story-bridge approach, the MHL program led to some improvement in participants’ knowledge about and attitudes toward people with mental disorders and was well received by the leaders. These studies suggest that using a MHL intervention program to target community and religious leaders is feasible and can be used in other community groups.

In light of the reviewed literature, the current study sought to evaluate the impact of a 6-h MHL workshop targeted towards Arabic speaking religious and community leaders based in South Western Sydney. This preliminary study aimed to evaluate if the training was successful in improving the recognition of PTSD related problems amongst refugees, knowledge regarding treatments for such problems; the reduction of negative attitudes towards people with PTSD and the promotion of professional help-seeking.

## Method

### Participants

Being a preliminary trial of a new culturally tailored MHL program, power analysis was undertaken to inform and guide future directions. Assuming training had a small effect size with a medium correlation between pre- and post-training scores, a sample size of 177 was identified as required. This number would give 95% power to detect a small effect size (d = 0.2) from pre- to post-training and follow-up with an alpha of 0.05. Alternatively, a sample size of n = 57 was identified as required if it was assumed that the training intervention would have a medium effect and made the conservative assumption that there would be no correlation between pre- and post-training scores. With these assumptions, this number would give 95% power to detect a medium effect size (d = 0.5) from pre- to post-training and follow-up with an alpha of 0.05.

A total of 54 participants were trained and 52 responded to the pre- and post-questionnaires. Participants were Arabic-speaking self-identified religious and other community leaders residing in South Western Sydney, Australia who represented a variety of organisations such as Churches/Mosques (n = 17), non-government (n = 21) and government organisation (n = 14). The training was promoted through religious centres and community networks including agencies that provide aid to humanitarian entrants in South Western Sydney. Participants were volunteers who made contact with the workshop coordinator (HL) for enrolment. Individuals were eligible to participate if they were from an Arabic background, self-identified as religious or other community leaders, had contact with Arabic speaking refugee groups on a permanent basis and had a good understanding of English language in order to complete the survey measures. Approval for this research was granted by the through South Western Sydney Health Local District Research (SWSLHD) Ethics Committee (reference number 2019/ETH12040) and joint approval with Western Sydney University (H13411).

### Intervention

The training intervention was a 6-h, classroom-style education program. It was developed by a working group that comprised of representatives from SWSLHD Health Promotion, New South Wales Transcultural Mental Health Centre, Western Sydney Local Health District (WSLHD), and Western Sydney University. The program was developed as a response to an identified need to improve the MHL of Arabic speaking religious and community leaders, an outcome emerging from a refugee mental wellbeing symposium held in SWSLHD in 2017; ‘Refugee Journeys From Surviving to Thriving’. This forum noted that working with Arabic speaking religious and community leaders was an immediate priority given the large numbers of Arabic speaking refugees resettling in the South Western Sydney area.

A culturally-sensitive program was designed by the working group and was based on evidence generated from previous research which had demonstrated a duality of treatment beliefs and preferences exists amongst refugee groups [11, 23, 24]. Recognising the importance of cultural and religious beliefs in collectivist societies such as Arab communities was considered essential to ensuring engagement and acceptability of the training by the target audience [25]. As such, care was taken to target areas of required knowledge such as recognition of mental health problems, treatment approaches utilised in Australia and challenging negative attitudes and stigma towards mental health problems, while ensuring this information was presented within a cultural valid framework. For example, when discussing depression and it’s symptoms, consideration of how it may present in Arabic speaking societies was highlighted. Moreover, when treatment approaches for depression utilising the biopsychosocial approach were discussed, the positive messages from religious teachings were respected as a valid enhancement to promote recovery for some. Further, when delivering knowledge on mental health systems in Australia, the content was interwoven with the role that community and religious leaders play in being the first point of contact and their ability to promote professional help-seeking amongst their community. The program was delivered in Arabic (orally) utilising multiple formats including PowerPoint presentations, video presentations and whole-group discussions to encourage interactive learning. In particular, videos highlighting the experiences of refugees and their mental health and featuring interviews with religious leaders were utilised because they demonstrated an excellent understanding of mental health and wellbeing. Teaching resources were developed to guide facilitation and ensure fidelity and the training was delivered by two bilingual Arabic speaking mental health clinicians (YM and RS) with significant expertise in transcultural mental health. The program was delivered in 2 sessions, breaking for morning and afternoon tea breaks and one longer lunch break. Table 1 presents the units taught.

**Table 1:** The structure and content of the training program

Morning session	Afternoon session
Topics presented: Health and wellbeing Social determinants of health Self-help strategies and how community and religious organisations can adopt the ‘5 Ways to Wellbeing’ framework The role of faith, spirituality and beliefs in wellbeing Stigma and risk factors Video (First clip) ‘Into the Light: An Arabic Resource on Psychological Health’.	Video (Second clip): ‘Into the Light: An Arabic Resource on Psychological Health’. Common mental health problems and their presentations in refugee populations Depression Posttraumatic Stress Disorder Help for mental health –navigating the mental health system in Australia

### Statistical analyses

The effectiveness of the training program was evaluated using an uncontrolled, repeated measures pre, and post design. Continuous variables were presented as means, whereas categorical variables are expressed as percentage (%) frequencies at pre-, and post-training points. McNemar and Wilcoxon Signed Ranks Tests were conducted to analyse continuous or binary outcome variables, as appropriate. A *p* value of less than 0.05 was considered indicative of statistical significance for all comparisons. Statistical data management and analyses of the data was carried out using Statistical Package for Social Sciences (SPSS 26.0 for Windows) [26].

### Measures

A self-report survey assessing key aspects of MHL modelled on the survey first reported by Jorm and colleagues [9] and further developed by the authors for refugee populations [11] was utilised. The survey was administered pre-intervention and immediately post-intervention. Socio-demographic characteristics of participants were also collected.

### Recognition of mental illness

Recognition of mental health problems was assessed using a culturally-valid vignette that described a fictional male Iraqi refugee named ‘Dawood’. Care was taken to ensure that the character met the criteria for PTSD as outlined in the 5th edition of the Diagnostic and Statistical Manual of Mental Disorders (DSM 5) [27] while avoiding the use of medical terminology. The use of vignettes in MHL research has been demonstrated to be ecologically valid [28, 29]. Following the presentation of the vignette, participants were asked in an open-ended format: `What, if anything, do you think is wrong with ‘Dawood’? Labels were coded as correctly identifying the ‘PTSD label category’ if they contained any of the following wording: ‘PTSD’; ‘post-traumatic stress disorder’; ‘post-trauma/tic stress/disorder’ and ‘PTS’. Researchers were also interested in examining recognition of a ‘general mental health problem’ and labels coded as correct for this category were ‘mental problem’, ‘mental illness’ and ‘mental disorder’.

#### Treatment knowledge: concordant, discordant and culturally informed treatment practices

Participants were next asked to rate the perceived helpfulness of different possible interventions—actions/activities, treatment providers and medications—for someone with Dawood’s problem. For the purposes of the evaluation, interventions classified into three categories, those that were concordant with evidence-based treatment approaches, those considered discordant or unhelpful, and those that had a culturally informed background. Interventions were classified as being concordant with evidenced-based treatment of PTSD using the framework developed by Reavley and colleagues [30]. These included the following: a typical family general practitioner (GP) or doctor; a psychologist; psychiatrist; becoming more physically active and improving diet; reading about people with similar problems and how they have dealt with them; relaxation, stress management and meditation; psychotherapy focused on thoughts and behaviours (cognitive behavioural therapy); and education/psychoeducation on the problem. Discordant or unhelpful items were ‘drinking alcohol to relax’ and ‘trying to deal with the problem alone’. Finally, items considered to comprise culturally informed care were: reading the Bible or Koran; having a prayer session with a clergy; talking with a clergy member; attending a social club of same cultural background; speaking with a close friend; and speaking with a family member. Scoring was undertaken by assigning one point for every intervention reported from the previous list which resulted in a total possible score out of eight for concordant interventions, out of two for discordant items and for cultural informed care, the total was out of six.

#### Negative attitudes towards mental illness

Participants’ negative attitudes towards those with mental illness were assessed using the modified Personal Stigma in Response to Mental Illness Scale [15, 28, 31]. Personal stigma was assessed by asking participants to respond to statements concerning the person described in the vignette using a 5-point Likert-type scale (1: ‘strongly disagree’ to 5: ‘strongly agree’). For purposes of analysis, the statements were divided into three components; ‘weak-not-sick’, ‘I would not tell anyone’ and ‘dangerous/unpredictable’ subscales, as previously used and validated [32]. The ‘weak-not-sick’ subscale focuses on the belief that the person is not ill and can control their behaviour (e.g. ‘Dawood could snap out of it if he wanted to’). The ‘I would not tell anyone’ subscale focuses on the belief it is better not to tell anyone about mental illness (e.g. ‘You would not tell anyone if you had a problem like Dawood’s). The ‘dangerous/unpredictable’ subscale focuses on the belief that someone with a mental illness is dangerous or unpredictable (e.g., ‘Dawood’s problem make him unpredictable’). Higher scores indicated greater personal stigma for each component. The ‘desire for social distance’ was assessed using five statements from the social distance scale developed by Link and colleagues [33], used in previous research by the authors [15]. Participants were asked to consider whether/to what extent they would be pleased to spend time with Dawood in different situations, for example ‘living next door to Dawood’ and ‘having Dawood marry into your family’. Responses to these items were scored on a 4-point Likert scale ranging from 1 (‘Yes, definitely’) to 4 (‘Definitely not’). A total social distance score was calculated as the sum of responses to the individual items, with higher scores indicating greater desire for social distance.

#### Providing support and helping advice

Participants were asked to ‘Describe all the things you would do to help Dawood’ using an open-ended format. De-identified responses were scored by a researcher (YM) who was blinded to whether they were collected at pre-, or post-training. A quality scoring system developed by the researchers was utilised to measure the quality of support and helping advice offered. Responses are scored across two categories, those that ‘promote appropriate help-seeking’, and those that promote ‘engaging with the person with the mental illness and offering support’. Specifically, a 1 point per item (up to maximum of 2 points) was awarded if the response mentioned encouraging Dawood to see a GP; psychologist; psychiatrist; social worker; community mental health services; and torture and trauma services such as NSW Service for the Treatment and Rehabilitation of Torture and Trauma Survivors (STARTTS). Similarly, 1 point per item (up to maximum of 2 points) was awarded if the response suggested engaging with the person in ways such as listening to the person; asking if they are OK; asking if they can help in any way; and offering practical assistance such as taking them to GP, helping with filling out a form; writing a letter of support; and calling other support services on their behalf. These components were included as they are deemed to be best practice and are recommended in the mental health care with refugee populations [34, 35]. A total possible score out of four was awarded with higher scores denoting higher quality of support and helping advice.

### Results

**Fig. 1:**
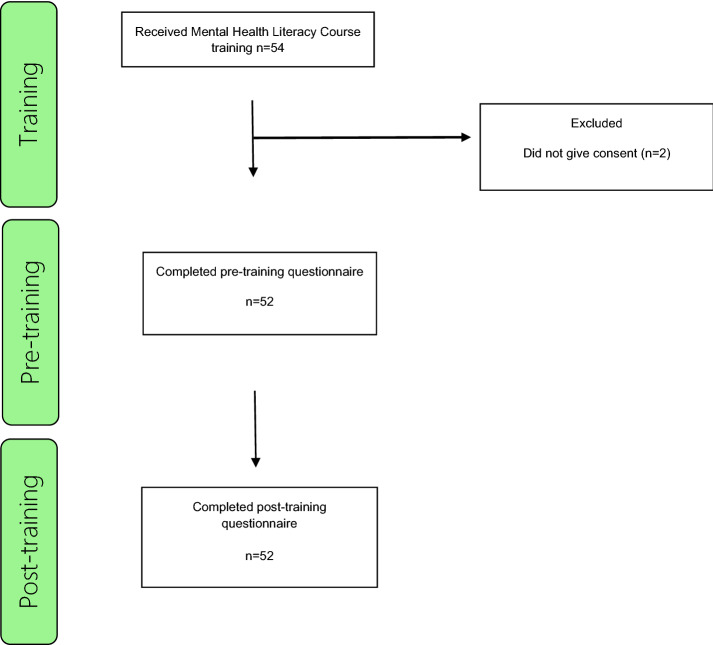
Flowchart of participants

Training workshops were held between September and October 2019. Figure 1 displays participant flow through the research stages. Demographic data on the participants are presented in Tables 2, 3.

**Table 2:** Socio-demographics characteristics of participants

Characteristics	Pre-training (n = 52)^a^	%
Gender		
Male	16	31
Female	36	69
Age (mean, SD)	47.06 (15.26)	–
Country of Origin (top 3)		
Iraq	18	35
Australia	13	25
Lebanon	8	15
Language spoken at home (top 3)		
Arabic	39	75
English	7	13.5
Assyrian	2	38.8
Marital status		
Never married	7	13.7
Married	35	68.6
Fiancée/partner	2	3.9
Divorced	6	11.8
Widowed	1	2
Education		
High school	3	5.8
Certificate	5	9.6
Diploma	5	9.6
Bachelor	31	59.6
Masters	6	11.5
For those born overseas		
Years in Australia (mean, SD)	17.60 (10.88)	–
Arrival status in Australia		
Refugee	7	19.4
Migrant	29	80.6

^a^May not add to 52 due to missing data

**Table 3:** Measures of Mental Health Literacy across time

Variables	Pre-training	Post-training	p-value (McNemar test)	p value (Wilcoxon signed-rank test)
Recognition of mental illness				
Problem recognised as ‘PTSD’ (%)	51	61.5	0.125	–
Problem recognised as ‘mental health problem’ (%)	62.7	80.8	0.035	–
Treatment knowledge				
Concordant treatment practices thought to be helpful (mean, SD)	6.38 (1.22)	6.73 (1.26)	–	0.061
Discordant treatment practices thought to be helpful (mean, SD)	0.13 (0.34)	0.13 (0.34)	–	1.0
Culturally informed treatment practices thought to be helpful (mean, SD)	3.96 (1.99)	4.39 (1.94)	–	0.086
Medications				
Treatment with antidepressant thought to be helpful (%)	60	82.4	0.000	–
Treatment with vitamins thought to be helpful (%)	55.1	54.9	1.0	–
Negative attitudes towards mental illness				
Personal stigma (mean, SD)				
Weak-not-sick subscale	1.88 (0.80)	1.86 (0.88)	–	0.480
I would not tell anybody subscale	1.98 (1.05)	1.75 (1.04)	–	0.111
Dangerous/unpredictable subscale	2.07 (0.79)	1.90 (0.79)	–	0.089
Desire for social distance (mean, SD)	9.31 (2.29)	8.62 (2.52)	–	0.042
Support and helping advice	1.90 (0.82)	2.24 (0.95)	–	0.032

#### Recognition of mental illness

In response to the question ‘What, if anything, do you think is wrong with ‘Dawood’?, 51% of participants correctly recognised the problem as ‘PTSD’ prior to the workshop which increased to 61.5% following the intervention, however, this increase was not significant (p = 0.125). To assess whether there was an increase in participants being able to recognise the problem described in the PTSD vignette as a ‘general mental health problem’ over time, the frequencies of all other responses that represented a mental health related label (‘mental illness’, ‘mental problem’, ‘mental breakdown’, ‘mental issue’) were included. Post-intervention there was a significant increase (p = 0.035), with the percentage reporting such labels increasing from 62.7 to 80.8%.

#### Treatment knowledge: concordant, discordant and culturally informed treatment practices

Participants knowledge regarding treatment deemed to be concordant with evidence-based treatment of PTSD did not significantly increase post-intervention (6.38 versus 6.73; p = 0.061). The endorsement of discordant treatment practices demonstrated floor effects with no change following training (0.13 versus 0.13; p = 1.0). Following the training, participants knowledge on culturally informed treatment practices also remained similar (3.96 versus 4.39; p = 0.086).

#### Medications

There was a significant increase in the endorsement of antidepressants as being helpful in the treatment of PTSD following training (60% initially versus 82.7%; p = 0.000).

#### Negative attitudes towards people with mental illness

There was a non-significant decrease in the belief that the character in vignette was ‘weak-not-sick’ from pre- to post-intervention (1.88 versus 1.86; p = 0.480). Additional non-significant decreases were observed in the ‘I would not tell anyone’ subscale from pre- to post-intervention (1.98 versus 1.75; p = 0.111) and in the ‘dangerous/unpredictable’ subscale from pre- to post-intervention (2.07 versus 1.90; p = 0.089). A statistically significant decrease was noted on the desire for social distance scale scores indicating participants were more willing to spend time with ‘Dawood’ following the intervention (9.31 versus 8.62; p = 0.042).

#### Support and helping advice

Following the intervention, there was a significant increase in participants scores on the quality of support and helping advice offered to the vignette character (1.90 versus 2.24; p = 0.032).

### Discussion

The current study sought to undertake a preliminary evaluation of a culturally tailored MHL intervention for Arabic speaking religious and community leaders of refugee communities residing in South Western Sydney, Australia. Using a pre and post study design, this pilot trial sought to measure if the intervention was effective in changing participants’ knowledge of common mental health problems in refugee populations, their attitudes towards those with mental health problems and advice provided to such individuals. A PTSD vignette based MHL survey was utilised to assess changes. Following the training, participants demonstrated a significant improvement in recognising the problem described in the vignette as a general mental health problem. and a greater understanding of the helpfulness of antidepressants in the treatment of PTSD. Further, post intervention, there was a reduction in the desire for social distance, a measure of negative attitudes, and the quality of helpful advice offered to those with a mental health problem also improved. However, not all measures demonstrated a significant improvement with knowledge of treatment approaches considered concordant with evidence-based treatment for PTSD increasing post-intervention but not significantly. Similarly, while recognition that the vignette described a person with PTSD increased, it did not reach statistical significance.

#### The treatment gap

This program was developed to respond to the evidence that posited religious and community leaders to be gatekeepers in refugee communities, providing mental health support and potentially facilitating professional help-seeking processes [18]. Relatedly, research has also demonstrated limited mental health service uptake amongst such individuals even when presenting with severe levels of psychological distress [5, 8]. Several factors have been postulated for this impaired help-seeking behaviour including negative perceptions of mental health treatment and the fear of being considered ‘crazy’ within their own community [18]. Such negative attitudes towards those with mental illness comprise an important aspect of the of MHL, which as previously stated has been demonstrated to influence professional help-seeking. Within the Arabic speaking refugee communities, MHL has been found to be problematic, including that of its community leaders [11, 12, 18]. Recognising this need, SWSLHD health promotion partnered with Arabic speaking mental health professionals, refugee mental health expert academics and transcultural health promotion experts in order to develop a culturally tailored intervention. Our aim was to deliver new knowledge on the mental health problems of refugees, mental health treatment approaches and Australian mental health systems; but to present this information respectfully alongside culturally and religiously informed practices such as seeking spiritual guidance and prayer. This dual stance recognises the importance of the client’s knowledge and was deemed necessary in order to engage the target participants. It also represents a point of difference from other training workshops on seeking to improve mental health knowledge.

#### Recognition of mental illness

Recognition of mental health problems have been found to be crucial in facilitating help-seeking [36]. It is argued that once individuals are able to recognise a particular mental health problem this can activate a *schema* about the appropriate action to take [36]. Thus our finding that training led to improved recognition the vignette described a ‘mental health problem’ is encouraging and necessary in order for leaders who are working with their community to better provide correct advice and guidance. This is even more so, because of the evidence that such leaders are likely to be the first point of contact in this community.

#### Treatment knowledge–the role of antidepressants

Another important finding was that participants knowledge on professionally-aligned and recognised interventions for managing PTSD such as the use of antidepressants [31] which was found to increase following training. Improved understanding of the role psychopharmacology may play in treatment of mental health disorders can lead to a reduction in negative attitudes associated with such treatment. This knowledge is important given that research has shown there is limited treatment awareness on the benefits for prescription medication in psychiatric care in the general public [37] and in ethnic minorities [38]. Further, increasing the understanding of the role antidepressants can play in treating some mental health disorders can have positive impacts on medication adherence through the action of community leaders, encouraging those they encounter in their community to use medication where it is needed.

#### Negative attitudes towards those with mental illness

Negative attitudes towards individuals with mental illness remains a significant problem in society despite considerable efforts to address these. Often, people with a mental illness report feelings of rejection brought on by fear-based exclusion processes such as the ‘not in my backyard’ response [33]. This can be even more pronounced in the Arabic speaking community [39]. As such, the finding that our intervention demonstrated a significant reduction in the desire for social distance, one measure of stigma towards those with mental illness (in this case PTSD) is heartening. It could be argued that achieving such changes in religious and community leaders can have a significant follow on effect by the virtue of the reach and influence such individuals hold. By taking a more positive view of interacting with those with mental illness, leaders are in better positions to respond in ways that will promote help-seeking rather potentially causing such individuals to hide for fear of shame and exclusion.

#### Support and helping advice

The quality of advice provided to a person with mental illness was also found to significantly improve. Post-intervention, participants described actions they would take to assist ‘Dawood’ which aligned with best practices in the mental health care with refugee populations [34, 35]. In particular, there was an increase in promoting professional help-seeking such as encouraging consulting with a GP or specialist refugee mental health services such as STARTTS. In addition, participants described useful approaches such as listening and asking if they are ‘OK’ and offering practical support such as writing letters of support and offering transportation to services.

#### Study limitations and strengths

A number of limitations should be noted. Firstly, not all the MHL measures demonstrated a statistically significant improvement post intervention. Notably, participants ability to recognise the vignette as describing a person with PTSD did not significantly improve despite the fact that PTSD was one of the mental health problems discussed in the training. Further, knowledge on concordant treatment approaches and measures of personal stigma did not significantly improve. Issues with curriculum design or the facilitation of the program may have contributed to the failure to find significant changes. However, arguably it is more likely to be related to the small sample size which limited statistical power to detect small effect sizes, particularly in light of the fact that all measures demonstrated *non*-*significant* improvement post intervention. Other limitations were the use of a quasi-experimental pre- post design which precluded assessment of changes over time and the recruitment of a convenience sample of volunteers limiting the current sample being representative of all Arabic speaking religious and community leaders. Using a PTSD based vignette to assess changes means that only recognition and attitudes towards PTSD were evaluated. This limitation could be addressed in future with a larger sample size that would allow for multiple mental illness vignettes being presented. Finally, there was no evaluation of the impact this training had on the actual mental health or help-seeking behaviours of Arabic speaking refugees that our leaders serve. In future, a larger sample size with a follow-up arm and incorporating qualitative measures on the curriculum content and its delivery would provide further insights. Additionally, the use of a control group who are provided matched non-mental health training such as education on physical health conditions and surveyed on such could allow for parsing out of training effects in general versus the content of our program. Strengths of this study include being the first program of its kind that aimed to improve MHL of Arabic speaking religious and community leaders using a culturally sensitive approach. Targeted areas of required knowledge such as recognition of mental health problems, treatment approaches utilised in Australia and challenging negative attitudes and stigma towards mental health problems were delivered alongside the recognition of the importance of cultural and religious beliefs. Our training served to complement the role of religious and community leaders and equip them with knowledge to serve as mental health allies promoting professional help-seeking where it was needed. Nonetheless, a recommended direction for future research would be to undertake a study using a Delphi methodology to further substantiate if culturally informed treatment approaches are recommended practices agreed to by a panel of experts in both refugee mental health along with community and religious leaders. The delivery of the program in Arabic by experienced mental health clinicians to ensure engagement and better comprehension was also considered a strength and likely to be a significant factor in the program being well-received. While there has been an increased emphasis on cultural competency in mental health care and the delivery of evidence-based psychosocial services for ethnic groups [34], to date, culturally-appropriate psychoeducation initiatives are limited and those directed towards community and spiritual leaders even more so. Finally, the processes and mechanism utilised in this study can potentially serve as a framework to shape culturally-appropriate MHL programs targeting leaders from other culturally-and-linguistically-diverse and or refugee groups in Australia.

## Conclusion

To the best of our knowledge, this is one of the first culturally sensitive programs focussed on improving the MHL of Arabic speaking community and religious leaders. Our findings suggested that the intervention was able to improve some measures such as the desire for social distance and the quality of support and advice provided to those with mental health problems. Recommended next steps should be tailoring and modifying the program by addressing the identified limitations and then undertaking a further roll out and evaluation of this training. In conclusion, this program represents the necessary first step needed to equip community leaders with the knowledge to better respond to mental health problems. As such the overall goal of improving the mental health outcomes of Arabic speaking refugee communities is closer to being realised.

## Abbreviations

ABS: Australian Bureau of Statistics
LGA: Local government area
MHFA: Mental health first aid
MDD: Major depression disorder
MHL: Mental health literacy
NSW: New South Wales
PTSD: Posttraumatic stress disorder
STARTTS: NSW Service for the Treatment and Rehabilitation of Torture and Trauma Survivors
SWSLHD: South Western Sydney Local Health District
SWSPHN: South Western Sydney Primary Health Network
UNHCR: United Nations High Commissioner for Refugees
WSLHD: Western Sydney Local Health District
